# Fatigue Life Prediction of CFRP-Strengthened RC Beams with Flexural Crack under Hygrothermal Environments

**DOI:** 10.3390/ma15134681

**Published:** 2022-07-04

**Authors:** Xinyan Guo, Hangyue Cui, Yilin Wang, Zhanbiao Chen

**Affiliations:** 1 School of Civil Engineering and Transportation, South China University of Technology, Guangzhou 510640, China; xyguo@scut.edu.cn (X.G.); 202120107677@mail.scut.edu.cn (H.C.); 2 Magnel–Vandepitte Laboratory, Department of Structural Engineering and Building Materials, Ghent University, Technologiepark—Zwijnaarde 60, 9052 Ghent, Belgium; yilin.wang@ugent.be

**Keywords:** CFRP, CFRP–concrete interface, failure mode, life prediction, RC beam

## Abstract

The durability of reinforced concrete (RC) beams strengthened with carbon fiber-reinforced polymer (CFRP) is a worldwide concern in structural engineering. As an important part of the strengthened beam, the performance of the CFRP–concrete interface under hygrothermal environments is a delicate problem. In this paper, the fatigue behavior of CFRP-strengthened RC beams is analyzed by a theoretical model. In the model, CFRP–concrete interface degradation under hygrothermal environments is involved. Since interface debonding and rebar fracture induced by intermediate cracking are two typical failure modes, the damage models of rebar and the CFRP–concrete interface are established. Based on the theoretical model, the failure mode of CFRP-strengthened RC beams can be predicted, and fatigue life can be determined. The results showed that IC debonding is more likely to occur under hygrothermal environments. The accurate prediction of failure modes is essential for fatigue life prediction.

## 1. Introduction

Existing reinforced concrete (RC) structures have become weak and dilapidated due to various factors, including vehicle growth, population explosion, and extreme environmental erosion. Fiber-reinforced polymer (FRP) composites have high tensile strength, low self-weight, and good corrosion resistance and have been extensively used to strengthen and rehabilitate RC bridge structures over the past decades [1,2,3].

There are considerable studies on the static behavior of FRP-strengthened RC beams [4,5,6]. In response to higher demand for sustained loads from pedestrians and vehicles, it is necessary to take into consideration the fatigue behavior of the strengthened beams. However, limited fatigue testing has been carried out [7,8,9]. B.G. Charalambidi [7] found that bonding FRP could provide the residual capacity of RC beams after rebar fracture, and the stiffness ratio of rebar to FRP was crucial to the fatigue behavior of FRP-strengthened beams. The fatigue life was controlled mainly by rebars. R. Al-Rousan [8] studied the fatigue behavior of CFRP-strengthened beams with nonlinear finite element analysis and found that the secant stiffness degraded due to fatigue loading. Stiffness degradation under fatigue loading is an important manifestation of cumulative fatigue damage. However, researchers assumed that there was a perfect bond between FRP and concrete, which was not inconsistent with the actual constructional engineering [7,8,9]. The failure modes of FRP-strengthened RC beams also include the debonding of the FRP–concrete interface. In E. Ferrier’s research [10], a slipping effect was involved in the RC beam section equilibrium that was analyzed by the delay characteristics of each constituent material. In particular, the relationship that exists between the strain and shear stress for the FRP–concrete interface was added to the theoretical model. The partly cohesive debonding in the concrete was the main reason for the failure of the RC beam. There was still a drawback in that the softening stage of the relationship between FRP and concrete was not involved. Therefore, a more appropriate bond–slip relationship should be used in this area.

The interfacial behavior of the FRP–concrete interface under monotonic loading has been investigated [11,12,13], and bond–slip models have been proposed [14,15,16,17]. Debonding failure of the FRP–concrete interface is regarded as one of the main failure modes after quasi-static loading. In comparison, the findings under fatigue loading are limited, such as the influence of concrete crack [18], stress distribution on FRP [19], and the bond–slip relationship of the FRP–concrete interface [19,20,21]. A. Al-Saoudi [18] simulated the cracking of concrete close to FRP under fatigue loading by FEA and proposed a crack softening law. The crack opening displacement was used as the criterion to judge the interface failure. H. Diab [22] incorporated Maxwell’s constitutive model into the FE model and found that the stress on FRP was redistributed due to the effect of fatigue loading, leading to an increase in the effective bond length with increasing loading cycles. K.Y.M. Loo [20] considered the bilinear bond–slip model in the FE model and assumed that the stiffness of the FRP–concrete interface was constant during the fatigue loading process. The bond strength was exhausted due to fatigue loading, which caused local debonding of the FRP–concrete interface. As a result, the peak stress under fatigue loading moved along the length until the peak stress under static loading could not be maintained. Since the fatigue failure models of FRP-strengthened RC beams include concrete crack propagation, rebar rupture, and interface debonding, the concrete crack length, damage to rebar and the FRP–concrete interface, and stiffness degradation should be considered in the failure criteria. Thus, it is quite significant to apply an appropriate theory to the failure criterion.

Bridges are serviced in various extreme environments, such as salt spray [23], hot and humid [24,25], freeze–thaw [26], ultraviolet radiation [27], and wet/dry circulation [28] environments, so it is necessary to study environmental effects on FRP-strengthened RC beams. The study of the FRP–concrete interface plays a significant role, because the interface is sensitive to environments. The epoxy resin of FRP composite is greatly influenced by temperature and humidity [29]. Zheng [30] carried out double-shear experiments on FRP–concrete interfaces under a simulated hygrothermal environment (60 °C, 95% R·H). The results indicated that the high-temperature and -humidity environment obviously decreased the interfacial stiffness. The influence of elevated service temperature (20, 50, 65, and 80 °C) on the bonding performance of the FRP–concrete interface was studied [31]. There was a relative decrease in fatigue performance with an increase in temperature. Additionally, it was found that the bond failure was characterized by a thin layer of concrete on FRP sheets under 50 and 65 °C but almost no concrete under 80 °C. Therefore, there are two interfacial failure modes: cohesive failure and adhesive failure. Adhesive failure tends to be predominant at high temperatures. To further analyze the interfacial failure criteria under elevated temperature, Dai [32] proposed the interfacial brittleness index and interfacial fracture energy determined by regression analysis of test data.

Finally, it is necessary to take into consideration the coupling effect of fatigue load and hygrothermal conditions on fatigue behavior of FRP-strengthened RC beams. Based on Huang’s results [33], Qin [34] established a fatigue life prediction model of FRP-strengthened beams subjected to fatigue load and a hygrothermal environment. The effects of temperature and relative humidity were considered as material coefficients, determined by regression analysis of test data. However, the critical part—the FRP–concrete interface—was not considered in these studies. Therefore, the failure mechanism of FRP-strengthened beams under fatigue load and a hygrothermal environment has not been clarified.

With a focus on the durability of CFRP-strengthened RC beams, a theoretical model considering the degradation of the CFRP–concrete interface under hygrothermal environments was proposed. With this model, the accumulated damage of strengthened beams with intermediate flexural crack was analyzed. According to the analysis, two main failure modes were considered: IC debonding and rebar fracture caused by the intermediate crack. Moreover, two corresponding failure criteria were established. The proposed theoretical model and calculation procedure can be used to predict the failure mode of strengthened beams and determine the fatigue life under hygrothermal environments.

## 2. Fatigue Life Prediction Model of Mid-Span Cracked Beam

During the fatigue process, the properties of RC beams degrade because of the concrete cumulative damage. RC beams are vulnerable to cracking because of the low tensile strength of concrete. In the preliminary studies of this research group [33,34,35], a typical failure mode of CFRP-strengthened RC beams was found which shows that one main type-I crack propagates with cyclic loads and finally leads to the failure. Therefore, this paper considered the situation of a CFRP-strengthened beam with an intermediate flexural crack under constant amplitude fatigue load. The mid-span deflection, shear stress distribution, and interfacial crack length changed as the loading cycle increased.

### 2.1. Interfacial Shear Stress Distribution

Several model types are suitable for the description of the interfacial bond–slip relationship, such as the bilinear, cutoff, elastic–plastic, and Popovics models [36]. Because of the good approximation of the interfacial non-linear relationship, the bilinear model is used in this paper. In the bilinear bond–slip model, three phases, including elasticity, softening, and debonding, are considered and presented:

(1) $$\tau \left(s\right)=\{\begin{array}{cc} k_{1}s; & 0<s\leq s_{0}\\ k_{2}\left(s_{u}-s\right); & s_{0}<s\leq s_{u}\\ 0; & s>s_{u} \end{array}\;$$

where s is the slip along the CFRP–concrete interface; $s_{0}$ is the interfacial slip corresponding to shear strength $\tau_{u}$; $s_{u}$ is the interfacial debonding slip; $k_{1}=\frac{\tau_{u}}{s_{0}}$ is the slope in the elastic phase; and $\;k_{2}=\frac{\tau_{u}}{s_{u}-s_{0}}$ is the slope in the softening phase.

**Figure 1:**
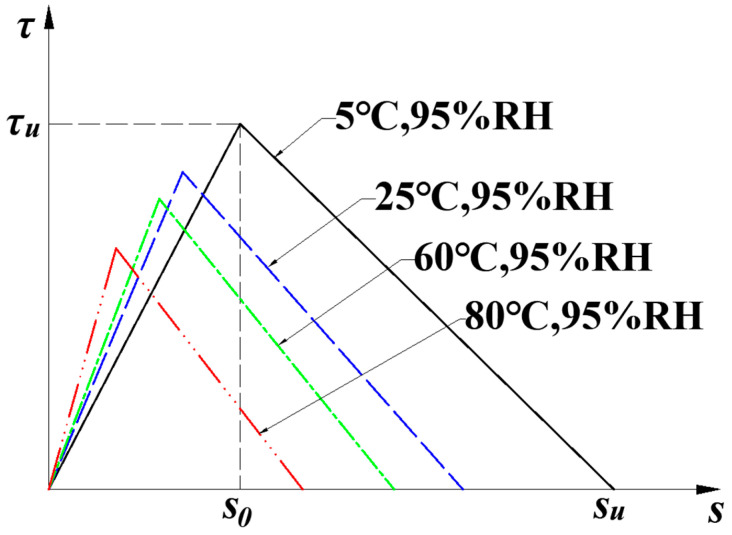
Bilinear bond–slip relationship with different hygrothermal pretreatments.

To study the influence of humidity and temperature variations on CFRP–concrete interface degradation, Zheng [37] conducted shear testing on concrete blocks externally bonded with CFRP. All specimens were exposed to an accelerated hygrothermal environment for 14 days. Degradation of the CFRP–concrete interface was significant compared to specimens without environmental pretreatment, especially with the effect of temperature. Bilinear bond–slip models were established with the tested bond parameters from Zheng’s results [37], as shown in Figure 1. The adapted parameters are listed in Table 1.

### 2.2. Time-Dependent Characters of Concrete

Because of its perfect fatigue resistance, CFRP has been considered a kind of linear elastic material in the whole fatigue process, but the concrete effective modulus *E_ci_* at the *i*th cycle degrades because of cumulative damage [38], which can be calculated by [39]:

(2) $$E_{ci}=\left(1-0.33\frac{n_{i}}{N_{ci}}\right)E_{c}$$

where *n_i_* is the cycle number; *E_c_* is the initial elastic modulus; and *N_ci_* is the fatigue life corresponding to compressive stress, which can be calculated by [39]:

(3) $$S_{ci}=0.9885-0.0618\mathrm{lg}N_{ci}$$

where *S_ci_* is the ratio of the compressive stress $\sigma_{ci}$ to the uniaxial strength *f_c_* at the *i*th cycle.

The main rebar in this paper is assumed to be elastoplastic. The rebar keeps linear elasticity before yielding, and when the tensile stress $\sigma_{s}$ is higher than the yield stress, it keeps constant at yield stress.

### 2.3. Properties at Mid-Span Section under Constant Amplitude Fatigue Load

**Figure 2:**
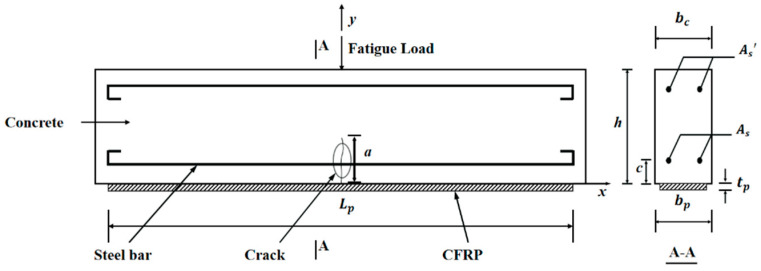
CFRP-strengthened RC beam with mid-span crack.

Figure 2 shows the typical cross-section character of a simple-supported CFRP-strengthened RC beam under cyclic fatigue load.

**Figure 3:**
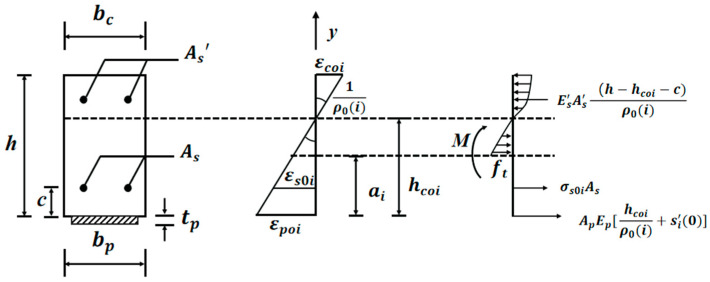
Distribution of stress-strain at the mid-span section.

Figure 3 shows the mid-span section of the strengthened beam under fatigue loading. As the number of cycles *i* increases, the degradation in concrete elastic modulus $E_{ci}$ causes many changes, such as crack length *a*_0*i*_, the neutral axis location *h_c_*_0*i*_, and the curvature *ρ*_0*i*_. Assuming that the cross-section strain is linear, Figure 3 shows the distribution of stress-strain at the mid-span section. The equilibrium equations can be established as follows:

(4) $$\{\begin{array}{c} A_{s}\sigma_{s0i}+b_{p}t_{p}E_{p}\left[\frac{\left(h_{c0i}\right)}{\rho_{0i}}+\delta_{i}^{\prime }\left(0\right)\right]+bE_{ct}\frac{{\left(h_{c0i}-a_{0i}\right)}^{2}}{2\rho_{0i}}=bE_{ci}\frac{{\left(h-h_{c0i}\right)}^{2}}{\rho_{i}}+A_{s}^{\prime }E_{s}^{\prime }\frac{\left(h-h_{c0i}-c\right)}{\rho_{i}}\\ A_{s}\sigma_{si}\left(h_{ci}-c\right)+b_{p}t_{p}E_{p}h_{c0i}\left[\frac{\left(h_{c0i}\right)}{\rho_{0i}}+\delta_{i}^{\prime }\left(0\right)\right]+bE_{ct}\frac{{\left(h_{ci}-a_{i}\right)}^{3}}{3\rho_{0i}}+bE_{ci}\frac{{\left(h-h_{c0i}\right)}^{3}}{2\rho_{0i}}+A_{s}^{\prime }E_{s}^{\prime }\frac{{\left(h-h_{c0i}-c\right)}^{2}}{\rho_{0i}}=M \end{array}$$

where *M* is the flexural moment; *b_p_* and *b* are the width of the CFRP and the beam, respectively; *A_s_* and *A_s_′* are the cross-section areas of tension- and compression-rebar, respectively; *h* is the height of the RC beam; *c* is the concrete cover thickness; *t_p_* is the thickness of the CFRP; and *E_p_*, *E_c_*_,_ and *E_s_′* are the elastic moduli of the CFRP, concrete in tension, and rebar in compression, respectively.

In Equation (4), the first derivative of the interface slips at mid-span $s_{i}^{\prime }\left(x\right)$ is established as follows:

(5) $$s_{i}^{\prime }\left(x\right)=\varepsilon_{pi}\left(x\right)-\varepsilon_{ci}\left(x\right)$$

where $\varepsilon_{pi}\left(x\right)$ and $\varepsilon_{ci}\left(x\right)$ are the strains of the CFRP and the concrete at the bottom of the RC beam, respectively.

Since the behavior of the CFRP–concrete interface is different in the elastic and softening phases, the interfacial slip and stress should be discussed separately. During the elastic stage, the interfacial stress τ does not reach $\tau_{u}$; then, the interfacial slip $\;s_{i}\left(x\right)$ is expressed as [35]:

(6) $$s_{i}\left(x\right)=A\left(e^{x\lambda \left(i\right)\sqrt{k_{1}}}+e^{-x\lambda \left(i\right)\sqrt{k_{1}}}\right)$$

where $\lambda \left(i\right)=\sqrt{\frac{1}{E_{p}\cdot t_{p}}+\frac{b_{p}}{E_{c}\left(i\right)\cdot b\cdot h}}$ and $A=\frac{-2\lambda \left(i\right)t_{p}\cdot \sigma_{p0i}\cdot e^{\lambda \left(i\right)\frac{L_{p}}{2}\sqrt{k_{1}}}}{\sqrt{k_{1}}\cdot \left(e^{\lambda \left(i\right)L_{p}\sqrt{k_{1}}}-1\right)}$.

Once τ reaches the maximum shear stress $\;\tau_{u}$, then the interface is in the softening stage. The interfacial slip $\;s_{i}\left(x\right)$ in the softening stage is:

(7) $$s_{i}\left(x\right)=B\cos\left[x\lambda \left(i\right)\sqrt{k_{2}}\right]+C\sin\left[x\lambda \left(i\right)\sqrt{k_{2}}\right]+s_{u}$$

*B* and *C* are constants which can be determined by boundary and continuous conditions [35].

At the tip of the intermediate crack, the tensile stress of concrete reaches uniaxial tensile strength $\;f_{ct}$:

(8) $$E_{ct}\frac{h_{c0i}-a_{i}}{\rho_{0i}}=f_{ct}$$

Because the tensile rebar is considered ideal elastoplastic, the expression of the tensile stress $\sigma_{s0i}$ at each cycle is:

(9) $$\sigma_{s0i=}\{\begin{array}{cc} E_{s}\varepsilon_{s0i}, & \varepsilon_{s0i}<\varepsilon_{y}\\ f_{y}, & \varepsilon_{s0i}>\varepsilon_{y} \end{array}$$

where *E_s_* is the elastic modulus; $\varepsilon_{y}$ is the yield strain; and $\;\varepsilon_{s0i}$ is the axial strain of the tensile rebar at the *i*th cycle, which can be given by:

(10) $$\varepsilon_{s0i}=\frac{\left(h_{c0i}-c\right)}{\rho_{0i}}$$

From the Equations (4)–(10), the intermediate crack length *a_i_*, the neutral axis height *h_c_*_0*i*_, and the curvature $\frac{1}{\rho_{0i}}$ can be determined. Meanwhile, at the *i*th cycle, the deflection *v*_0*i*_ at mid-span can be expressed as:

(11) $$v_{0i}=\frac{PL^{3}}{48M\rho_{i}\left(0\right)}$$

### 2.4. Failure Criterion

Fatigue tests on CFRP-strengthened RC beams have been carried out in preliminary research [40,41,42]. Results showed that there were several failure modes, including tensile rebar fracture, CFRP–concrete interface debonding, and compressive concrete crushing. Tensile rebar rupture at mid-span and CFRP–concrete interface debonding always occurred, especially under hygrothermal environments. Moreover, these two failure modes may occur simultaneously. However, which failure will occur first is difficult to predict. Therefore, a fatigue life prediction method considering failure modes is discussed in this section.

#### 2.4.1. Cumulative Damage Model of Rebar

As described in Section 2.3, with the increase in loading cycles, the tensile stress of rebar increases because of the modulus degradation of concrete. A typical *S–N* relationship [43] of rebar is involved:

(12) $$\log N_{i}=\{\begin{array}{c} \begin{array}{c} \begin{array}{c} \begin{array}{cc} 8.460-0.011S_{si}, & 420.5<S_{si}\leq 787.7 \end{array}\\ \begin{array}{cc} 47.96-0.105S_{si}, & 411.0<S_{si}\leq 420.5 \end{array} \end{array}\\ \begin{array}{cc} 7.578-0.006S_{si}, & 251.6<S_{si}\leq 411.0 \end{array} \end{array}\\ \begin{array}{cc} 10.29-0.017S_{si}, & 192.2<S_{si}\leq 251.6 \end{array} \end{array}$$

where *S_si_* is the amplitude of applied tensile stress in MPa and *N_i_* is the fatigue life of rebar corresponding to *S_si_*.

By using Miner’s rule, the cumulative damage of rebar $D_{s}$ can be calculated:

(13) $$D_{s}=\sum^{\;}\frac{n_{i}}{N_{i}}$$

where *n_i_* is the cycle number of rebar at the stress amplitude $S_{si}$.

Based on Equations (9) and (10), $S_{si}$ can be calculated at peak and valley loads. During the computing process, when the cumulative damage $D_{s}$ reaches 1, the rebar fractures.

#### 2.4.2. Cumulative Damage of CFRP–Concrete Interface

The debonding failure of strengthened RC beams can be divided into two types: intermediate crack (IC) failure and plate end (PE) failure. Due to the existence of strain difference at the CFRP end, PE failure occurs after the separation of CFRP from concrete. Meanwhile, IC debonding failure initiates from the intermediate crack at the mid-span of the beam; this is because the higher interfacial shear stress is around the intermediate crack [44].

PE debonding failure needs to be considered when there is no intermediate crack. To avoid such a debonding failure, it is effective to anchor the end of the CFRP. However, flexural cracks always appear at the mid-span of strengthened RC beams, which increases the possibility of IC debonding failure. Thus, IC debonding failure needs to be involved in this study.

The energy release rate in the CFRP–concrete interface under the flexural moment $G_{R}$ can be expressed as [45]:

(14) $$G_{R}=\frac{1}{b_{p}}\left[\left|\frac{\partial W_{ext}}{\partial a}\right|-\left|\frac{\partial W_{sys}}{\partial a}\right|\right]$$

where $\frac{\partial W_{ext}}{\partial a}$ is the changing rate of the work performed by external force; $\frac{\partial W_{sys}}{\partial a}$ is the changing rate of the energy dissipated caused by moment-curvature and CFRP strain; and ∂a is the length increment of the interfacial crack.

In Equation (15), the changing rate of the work performed by external force is:

(15) $$\frac{\partial W_{ext}}{\partial a}=\frac{P\Delta v}{\Delta a}$$

where *P* is the external load; Δa is the length increment of the interfacial crack; and Δv is the deflection increment at the loading point.

The changing rate of dissipated energy $\frac{\partial W_{sys}}{\partial a}\;$ includes the work performed on the CFRP ($\Delta W_{p}$) and on the RC beam ($\Delta W_{b}$).

(16) $$\Delta W_{b}=\frac{1}{2}\left(M_{1a}+M_{2a}\right)\left(\frac{1}{\rho_{2}\left(x\right)}-\frac{1}{\rho_{1}\left(x\right)}\right)$$

(17) $$\Delta W_{p}=\frac{1}{2}b_{p}t_{p}\left(\sigma_{1p}+\sigma_{2p}\right)\left(\varepsilon_{2p}-\varepsilon_{1p}\right)$$

where the subscripts 1 and 2 represent the states before and after the debonding crack of the CFRP–concrete interface, respectively; $M_{1a}$ and $M_{2a}$ are the corresponding flexural moments; $\frac{1}{\rho_{2}\left(x\right)}$ and $\frac{1}{\rho_{1}\left(x\right)}$ are the corresponding curvatures; $F_{1p}$ and $F_{2p}$ are the corresponding CFRP tensile forces; and $\varepsilon_{2p}\;$ and $\varepsilon_{1p}$ are the corresponding CFRP tensile strains.

If $G_{R}\geq G_{f}$, IC debonding failure occurs according to the failure criteria. The failure fracture $G_{f}$ is different under various hygrothermal environments, and the values are listed in Table 1.

### 2.5. Calculation Procedure

**Figure 4:**
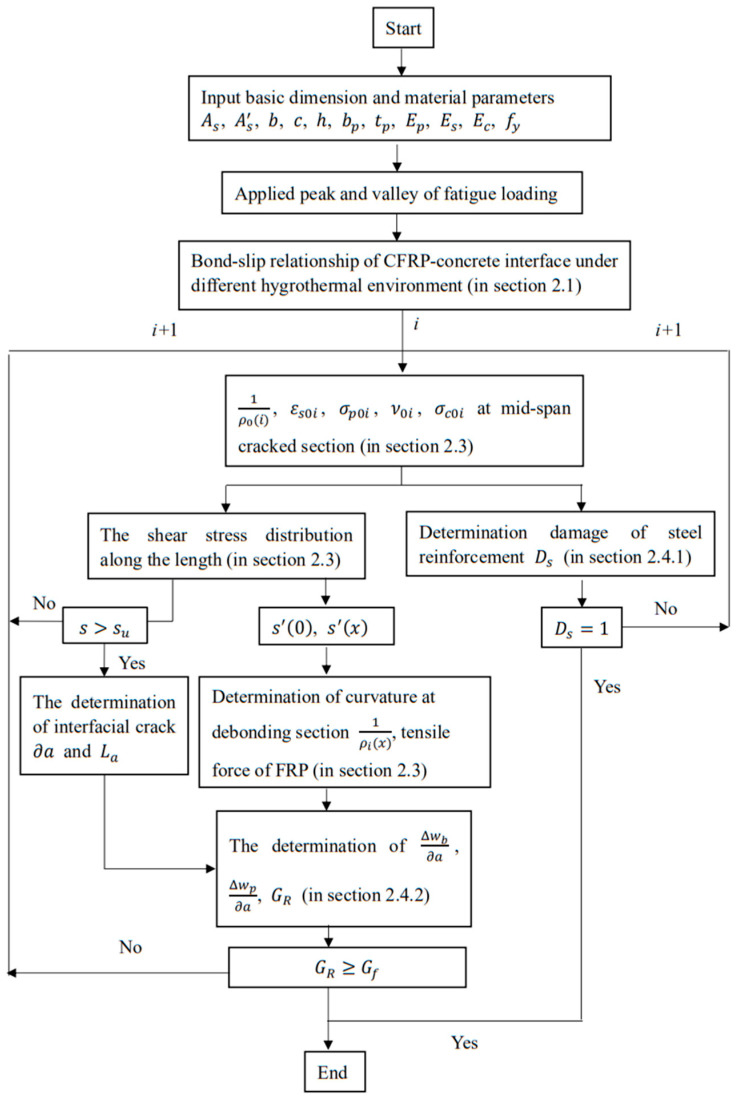
Flowchart for calculation procedure.

Based on the prediction model, the fatigue life of CFRP-strengthened RC beams can be calculated. Figure 4 describes the implementation steps for predicting failure modes and the corresponding fatigue life. The details are as follows:

The dimensions and material parameters of the strengthened beam are inputted into the theoretical model.

The bond–slip relationships under different environments and fatigue loads are inputted into the theoretical model.

The peak and valley values of cyclic loads are inputted into the theoretical model. From the previous step, the degraded elastic modulus and compression stress of concrete can be obtained by Equations (2) and (3). The parameters of the concrete after degradation are entered in the next step.

The curvature $\frac{1}{\rho_{0i}}$, the rebar stress $\sigma_{s0i}$, the deflection $v_{0i}$, and the concrete compressive stress $\sigma_{c0i}$ at the intermediate cracked section can be determined in each step (Section 2.3).

In each step, the cumulative damage to rebar $D_{s}$ (Section 2.4.1) can be obtained. If $D_{s}$ reaches 1, rebar rupture will be the main failure mode, and the corresponding loading cycle number *i* will be the predicted fatigue life.

The shear stress distribution can be obtained (Section 2.1). When the interfacial bonding slip reaches $s_{u}$, the interfacial crack will propagate. Then, the interface crack increment ∂a can be obtained.

From the theory involved in Section 2.3, $\frac{\Delta W_{b}}{\partial a}$, $\frac{\Delta W_{p}}{\partial a}$, and $G_{R}$ are determined.

If $G_{R}\geq G_{f}$, the failure mode will be IC debonding, and the corresponding loading cycle number *i* will be the predicted fatigue life.

As long as either one of the cumulative damages, $D_{s}\;$ or $D_{b}$, reaches 1, the corresponding failure mode will occur first.

## 3. Results and Discussions

To verify the effect of the hygrothermal environment on strengthened beams’ fatigue performance, numerical examples for strengthened beams were proposed. At a constant relative humidity of 95%, temperatures varied from 5 °C to 80 °C. Then, the distribution of interfacial stress and failure mode changed accordingly. The predicted fatigue lives corresponding to each failure mode were calculated.

The major parameters of bond–slip relationships with and without hygrothermal pretreatments are listed in Table 1. Regarding the numerical calculations, the parameters of materials and geometric features listed in Table 2 and Table 3 are similar to those used by Qin [34] and Wang [43]. Under hygrothermal environments, the failure mode of rebar rupture or IC debonding can be predicted according to the failure criteria. Moreover, the corresponding fatigue lives of strengthened beams were also predicted and compared with the test results.

### 3.1. Deflection of the Mid-Span Section

**Figure 5:**
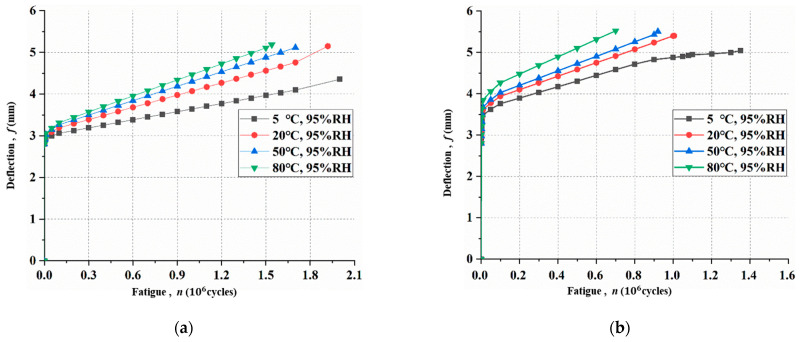
Maximum deflections under different fatigue loads and hygrothermal environments: (**a**) 27.5 kN; (**b**) 30.0 kN.

Figure 5 shows the deflections versus loading cycles before $D_{s}$ reaches 1 under different hygrothermal environments. The maximum deflection increases with increasing temperature. The reason for stiffness degradation is that the slope for the bond–slip relationship at the interface changes when exposed to various hygrothermal environments. For specimens at a peak fatigue load of 27.5 kN (Figure 5a), the corresponding maximum mid-span deflections under hygrothermal environments of 5 °C, 20 °C, 50 °C, and 80 °C are 4.35 mm, 4.65 mm, 5.35 mm, and 5.62 mm, respectively. For specimens at a peak fatigue load of 30.0 kN (Figure 5b), the corresponding maximum mid-span deflections under hygrothermal environments of 5 °C, 20 °C, 50 °C, and 80 °C are 4.85 mm, 5.39 mm, 5.35 mm, and 6.15 mm, respectively. Moreover, under the same hygrothermal environment, the maximum deflection increases with the increase in peak fatigue load.

### 3.2. Cumulative Damage of Rebar

**Figure 6:**
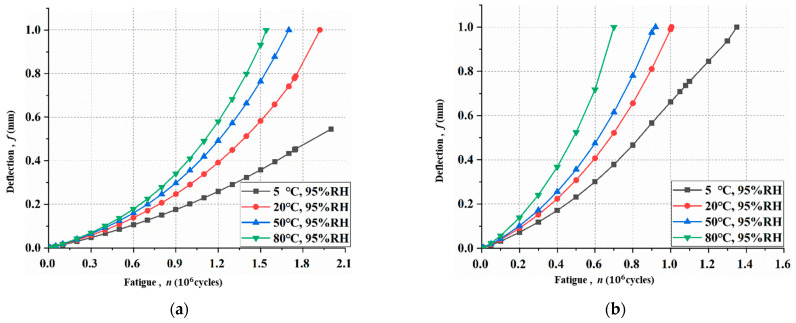
Cumulative damage of rebar under different hygrothermal environments at different loading levels: (**a**) 27.5 kN; (**b**) 30.0 kN.

Based on the calculation procedure, the tensile stress and cumulative damage of rebar $D_{s}$ at different cycles can be derived by Equations (12) and (13). The cumulative damage of rebar $D_{s}\;$ versus loading cycles under different hygrothermal environments is shown in Figure 6. As shown in Figure 6a, $D_{s}$ did not reach 1 at two million loading cycles under hygrothermal conditions of 5 °C, 95% R·H. With the increase in temperature, $D_{s}$ grows rapidly because of the increase in mid-span deflection caused by the effect of the hygrothermal environment. Furthermore, it was also observed from Figure 6 that the $D_{s}$ increased with the increase in fatigue loading level.

### 3.3. Shear Stress Distribution

**Figure 7:**
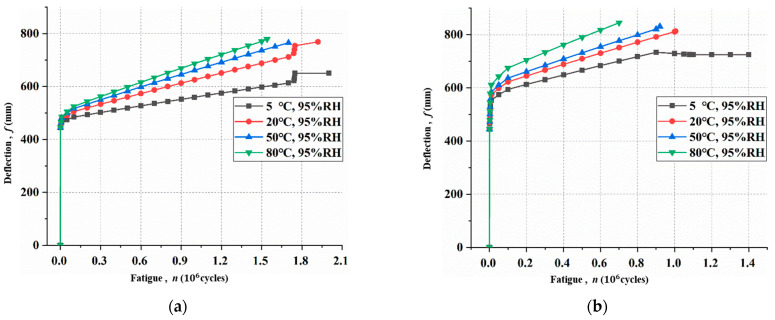
The tensile stress of CFRP under different hygrothermal environments at different loading levels: (**a**) 27.5 kN; (**b**) 30.0 kN.

Figure 7 shows the mid-span deflections versus loading cycles under different hygrothermal environments before $D_{s}$ reaches 1. Under the same environment, the tensile stress of CFRP at the mid-span section increases as loading level increases. This phenomenon is due to the elastic-plastic characteristics of rebar and intermediate crack propagation; CFRP should endure more loads.

Under the same loading level, the CFRP tensile stress increases with the temperature increase. At the loading level of 27.5 kN, the tensile stress of CFRP at 80 °C, 95% R·H increased by 33% compared with that at 5 °C, 95% R·H. When the loading level was 30 kN, the tensile stress of CFRP at 80 °C, 95% R·H increased by 21% compared with that at 5 °C, 95% R·H when $D_{s}$ reached 1. The calculation results show that the high-temperature and -humidity environment has a significant effect on the tensile stress of CFRP, which is mainly due to its great influence on the bond behavior.

**Figure 8:**
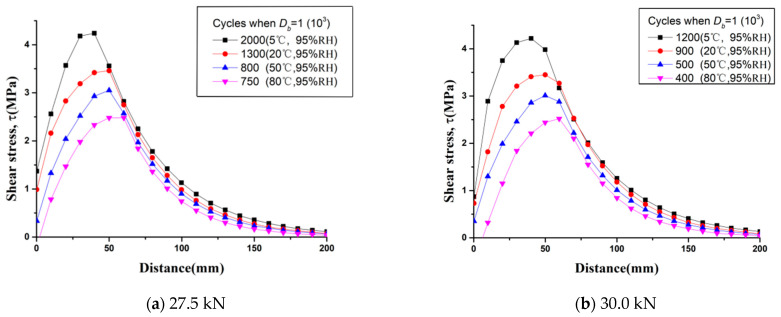
Shear stress distribution of CFRP–concrete interface in theory when *D_b_* = 1: (**a**) 27.5 kN; (**b**) 30.0 kN.

Based on the calculation procedure, the shear stress distribution τ and the energy release rate $G_{R}$ of the CFRP–concrete interface can be obtained. Figure 8 shows the shear stress distribution along with the CFRP–concrete interface under different hygrothermal environments when *D_b_* reaches 1. In Figure 8, the mid-span section with intermediate bending crack is at the location of *x* = 0. The length from the mid-span with an interfacial shear stress of 0 is the debonding length and denoted by $\;L_{a}$. Results show that the shear stress of the interface beyond 200 mm is 0 when *D_b_* reaches 1, and so the interfacial shear stresses beyond 200 mm have not been plotted.

With the comparison between the cumulative damages of rebar and the CFRP–concrete interface, the failure mode of the strengthened beam can be determined. The predicted failure modes and predicted lives of all specimens, corresponding to *D_s_* = 1 and *D_b_* = 1, are listed in Table 4 below.

For a strengthened beam under a loading level of 27.5 kN, the cycle number when *D_b_* = 1 decreased obviously with the increase in temperature. *D_s_* reached 1 before *D_b_* at 5 °C, 95% R·H, which means that the corresponding failure mode was rebar rupture. However, when the temperature reached 20 °C or above at 95% R·H, *D_b_* reached 1 first, and the corresponding failure mode was IC debonding. In the case of a strengthened beam tested under a loading level of 30.0 kN, two failure modes (rebar fracture and IC debonding) occur simultaneously at 5 °C, 95% R·H. *D_b_* reached 1 first under other environments, and the corresponding failure mode was IC debonding. The research results show that CFRP-strengthened RC beams subjected to fatigue loading with a high loading level are more likely to fail with IC debonding under hygrothermal environments.

## 4. Experimental Validation

Fatigue tests were conducted on the CFRP-strengthened RC beams to verify the proposed theoretical model and calculation procedure. The three-point bending fatigue tests were performed with a MTS-810 testing system. During the fatigue tests, the force-control mode with sine wave was adopted. In addition, the loading frequency was set at 10 Hz, while the stress ratio was 0.2. All RC beams were prepared in accordance with the code of practice. The material parameters and configuration of the CFRP-strengthened RC beam are listed in Table 2 and Table 3 and are the same as the example in Section 2. The epoxy resin adhesive was Lica-301, which was also used by Zheng [30]. The bond–slip properties under different hygrothermal environments are listed in Table 1.

A total of 26 specimens were prepared and divided into eight groups, which were subjected to different loading levels and hygrothermal environments. Before carrying out the fatigue tests, each group of specimens was exposed to simulated environments for 14 days at 5 °C, 95% R·H; 20 °C, 95% R·H; 50 °C, 95% R·H; and 80 °C, 95% R·H, respectively. According to a preliminary study of this group [34], the static load-bearing capacity $P_{u}$ of the same specimen was 41.7 kN. The peak load *P*_max_ was set at 27.5 kN (66% of $P_{u}$) and 30.0 kN (72% of $P_{u}$). Table 4 presents the details of all of the specimens.

**Figure 9:**
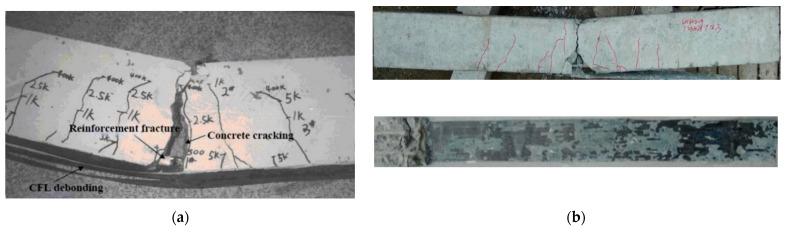
Failure modes of CFL-strengthened RC beam: (**a**) rebar fracture; (**b**) IC debonding failure.

Typical failure modes are shown in Figure 9. It can be seen that flexural concrete cracks appeared at the beginning of the fatigue test. Most cracks grew rapidly at the first stage (N ≤ 5000) and remained stable when they reached the neutral axis. The main crack at the mid-span section continued to propagate, which caused larger damage accumulated of the tensile rebars. Similar results and conclusions were also found in the environmental fatigue tests conducted on the same strengthened beams [46].

**Figure 10:**
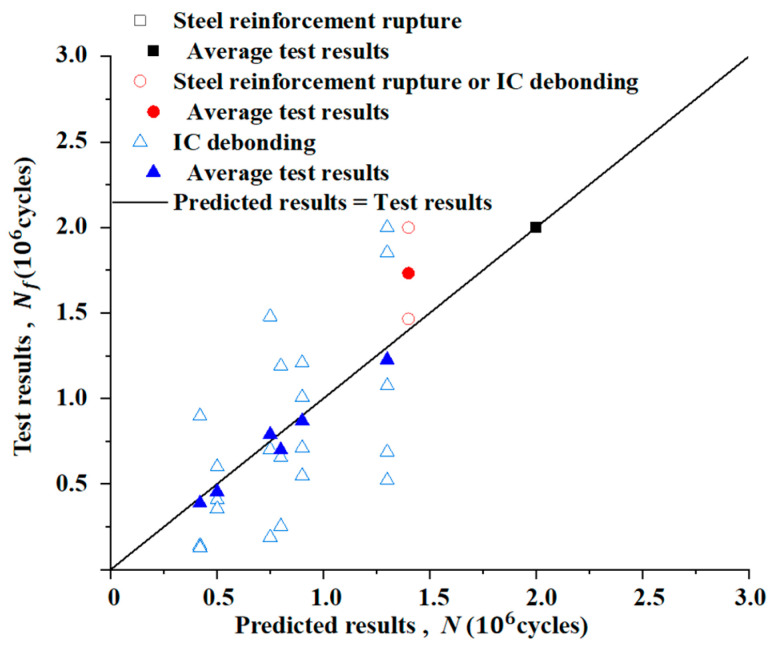
Comparison of the tested and predicted fatigue lives.

According to the proposed theoretical model, it is possible to predict the fatigue life of strengthened beams subjected to different loading and environmental conditions. Both the tested and predicted lives are shown in Figure 10. It should be noted that values greater than 2 × 10^6^ are treated as 2 × 10^6^. Although the fatigue lives at a loading level of 27.5 kN are dispersed because of individual differences, the average lives match well with the predicted results. The maximum relative error is 19.2% compared with the average test results. For the fatigue lives at a loading level of 30 kN, the relative error is 4.2% in the case of IC debonding failure. However, there is a large relative error of 63% in the case of rebar rupture. Therefore, the failure of IC debonding should be taken into consideration when CFRP-strengthened RC beams are subjected to high-level fatigue loads.

## 5. Conclusions

A fatigue life prediction model for CFRP-strengthened RC beams under hygrothermal environments was proposed, with consideration of the interfacial characters of CFRP–concrete. Corresponding failure criteria for rebar fracture and IC debonding were established. The proposed model can be used to analyze the fatigue behavior of strengthened beams and provide a scientific basis for life evaluation and retrofit design of RC bridges. The following conclusions have been drawn:

Flexural concrete cracks grew rapidly and remained stable, while the main crack at the mid-span section continued to propagate and caused damage accumulated in rebars. The performance of concrete and the CFRP–concrete interface degraded under fatigue loads and hygrothermal conditions, leading to increases in crack length and deflection.

The behavior of the CFRP–concrete interface degraded significantly under hygrothermal environments. When the strengthened beam was subjected to low-temperature conditions, the test failure mode was rebar rupture, which agreed with the model prediction. In the case of high-temperature conditions, the failure mode changed to IC debonding, which was also successfully predicted.

The interfacial shear stress exhibited a large increase under a high loading level, leading to IC debonding failure. The accuracy of fatigue life prediction was enhanced with the consideration of failure modes.

Fatigue life prediction matched the average test results well, but there were errors caused by specimen discreteness. Further research will be focused on FEM simulation, which will be helpful for improving the proposed model.

## Figures and Tables

**Table 1:** Major bond parameters for bond–slip relationship with different hygrothermal pretreatments (data from [37]).

Hygrothermal Conditions	$\tau_{u}$ (MPa)	$s_{0}$ (mm)	$s_{u}$ (mm)	$k_{1}$	$k_{2}$	$G_{f}$
5 °C, 95% R·H	4.25	0.055	0.266	77.3	15.9	0.515
20 °C, 95% R·H	3.46	0.036	0.174	96.1	19.9	0.176
50 °C, 95% R·H	3.05	0.030	0.143	101	21.3	0.094
80 °C, 95% R·H	2.53	0.019	0.096	133	26.4	0.054

**Table 2:** Parameters of RC beam strengthened by CFRP.

$E_{p}$ **(GPa)**	$E_{c}$ **(GPa)**	$E_{s}$ **(GPa)**	$E_{s}^{\prime }$ **(GPa)**	$f_{c}$ **(MPa)**	$f_{ct}$ **(MPa)**	$f_{y}$ **(MPa)**
230	34	206	206	47.6	4.45	400

**Table 3:** Geometric features of RC beam strengthened by CFRP.

*b* (mm)	*h* (mm)	*L* (mm)	$L_{p}$ (mm)	$t_{p}$ (mm)	$b_{p}$ (mm)	*c* (mm)	$A_{s}$ (mm^2^)	$A_{s}^{\prime }$ (mm^2^)
100	200	1850	1560	0.23	100	30	157	100

**Table 4:** Detailed test information and prediction results.

Hygrothermal Environment	Specimen No.	Tested Life, *N_f_*	Average Life, *N_f,_*_ave_	Predicted Failure Mode	Predicted Life (*D_s_* = 1)	Predicted Life (*D_b_* = 1)
5 °C 95% R·H	A5-27.5-1	>2 × 10^6^	>2 × 10^6^	A	>2 × 10^6^	>2 × 10^6^
	A5-27.5-2	>2 × 10^6^				
	A5-30.0-1	1,465,930	1,732,965	A and B	1,400,000	1,400,000
	A5-30.0-2	>2 × 10^6^				
20 °C 95% R·H	B20-27.5-1	1,853,118	1,227,000	B	1,920,000	1,300,000
	B20-27.5-2	521,580				
	B20-27.5-3	>2 × 10^6^				
	B20-27.5-4	686,534				
	B20-27.5-5	1,073,814				
	B20-30.0-1	1,007,974	869,046	B	1,050,000	900,000
	B20-30.0-2	1,208,013				
	B20-30.0-3	711,981				
	B20-30.0-4	548,219				
50 °C 95% R·H	C50-27.5-1	654,546	699,349	B	1,760,000	800,000
	C50-27.5-2	1,190,746				
	C50-27.5-3	252,756				
	C50-30.0-1	407,828	454,307	B	960,000	500,000
	C50-30.0-2	600,001				
	C50-30.0-3	355,094				
80 °C 95% R·H	D80-27.5-1	188,882	790,312	B	1,600,000	750,000
	D80-27.5-2	702,624				
	D80-27.5-3	1,479,430				
	D80-30.0-1	140,175	388,166	B	700,000	420,000
	D80-30.0-2	127,738				
	D80-30.0-3	896,585				

Note: the first part of Specimen No. refers to the environment group; the second is the peak load, and the third is the serial number. Failure mode A refers to rebar rupture and B refers to IC debonding failure.

## Data Availability

The data presented in this study are available on request from the corresponding author.
